# Polygenic Adaptation and Clonal Interference Enable Sustained Diversity in Experimental *Pseudomonas aeruginosa* Populations

**DOI:** 10.1093/molbev/msab248

**Published:** 2021-08-19

**Authors:** Katrina B Harris, Kenneth M Flynn, Vaughn S Cooper

**Affiliations:** 1 Department of Microbiology and Molecular Genetics, and Center for Evolutionary Biology and Medicine, University of Pittsburgh, Pittsburgh, PA, USA; 2 Department of Molecular, Cellular, and Biomedical Sciences, University of New Hampshire, Durham, NH, USA

**Keywords:** soft sweep, experimental evolution, parallelism, bacterial adaptation

## Abstract

How biodiversity arises and can be maintained in asexual microbial populations growing on a single resource remains unclear. Many models presume that beneficial genotypes will outgrow others and purge variation via selective sweeps. Environmental structure like that found in biofilms, which are associated with persistence during infection and other stressful conditions, may oppose this process and preserve variation. We tested this hypothesis by evolving *Pseudomonas aeruginosa* populations in biofilm-promoting arginine media for 3 months, using both a bead model of the biofilm life cycle and planktonic serial transfer. Surprisingly, adaptation and diversification were mostly uninterrupted by fixation events that eliminate diversity, with hundreds of mutations maintained at intermediate frequencies. The exceptions included genotypes with mutator alleles that also accelerated genetic diversification. Despite the rarity of hard sweeps, a remarkable 40 genes acquired parallel mutations in both treatments and often among competing genotypes within a population. These incomplete soft sweeps include several transporters (including *pitA, pntB, nosD*, and *pchF*) suggesting adaptation to the growth media that becomes highly alkaline during growth. Further, genes involved in signal transduction (including *gacS*, *aer2, bdlA*, and PA14_71750) reflect likely adaptations to biofilm-inducing conditions. Contrary to evolution experiments that select mutations in a few genes, these results suggest that some environments may expose a larger fraction of the genome and select for many adaptations at once. Thus, even growth on a sole carbon source can lead to persistent genetic and phenotypic variation despite strong selection that would normally purge diversity.

## Introduction

Bacterial populations inhabit countless environments along a continuum of spatial structure, ranging from a well-mixed liquid to rugged, solid surfaces. Growth on surfaces is associated with biofilm production that in turn generates nutrient and oxygen gradients (Stoodley et al. 2002; Dietrich et al. 2013), but so too does the metabolic activities of neighboring cells. Life in confined spaces results in varied levels of nutrients, waste, and signaling molecules that may alter selective forces on parts of the population and create novel ecological opportunities (Poltak and Cooper 2011). These physical differences are hypothesized to select for mutations in biofilm populations distinct from those in well-mixed cultures, which is supported by experimental microbial evolution studies (Traverse et al. 2013; Santos-Lopez et al. 2019). The greater environmental structure of the biofilm that effectively subdivides populations also can increase genetic diversity, for example, enabling phenotypically similar mutants to coexist when only the best (or luckiest) would prevail in well-mixed environments (Davey and O’Toole 2000; McElroy et al. 2014). Over longer time scales, this greater diversity increases competition between adaptive mutations, also known as clonal interference (Rainey and Travisano 1998; Boles et al. 2004; Habets et al. 2006; Wong et al. 2012; Traverse et al. 2013; Flynn et al. 2016; Santos-Lopez et al. 2019). Despite this process of diversification, replicate populations propagated in both biofilm and planktonic conditions exhibit high levels of both phenotypic and genetic parallelisms, suggesting some measure of predictability within the same environment (Wong et al. 2012; Tognon et al. 2017; Yen and Papin 2017; Sanz-García et al. 2018; Turner et al. 2018). We still have much to learn about how the biofilm life cycle influences evolutionary dynamics and processes, including the relative roles of mutation and selection, and whether biofilm growth becomes the dominant selective force relative to other stresses like nutrient limitation or external toxins.

*Pseudomonas aeruginosa* is an opportunistic pathogen found in soil and water and is known for its ability to thrive in numerous environments. *P. aeruginosa* is highly studied due to its association with poor outcomes in clinical settings where it forms biofilm-associated infections within patients (Eichner et al. 2014; Feliziani et al. 2014; Schick and Kassen 2018; Gloag et al. 2019). Adaptation to biofilm growth has been indicated as a cause of infection persistence in numerous studies (Parsek and Singh 2003; Periasamy and Kolenbrander 2009; Yildiz and Visick 2009). This adaptation manifests as important colony phenotypes such as mucoidy and rugose small colony variants (RSCVs) as well as the loss of virulence factor production and altered cell surface virulence determinants (Ashish et al. 2013; Kim et al. 2014). Several studies have been performed to identify some of the first steps of adaptation to a biofilm environment, but these studies have yet to replicate the high levels of genetic diversity often seen in chronic biofilm-associated infections, such as those of the airways of cystic fibrosis patients (Lieberman et al. 2011; Hogardt and Heesemann 2013; Marvig et al. 2015; Kruczek et al. 2016; Schick and Kassen 2018).

We previously conducted a long-term evolution experiment over 90 days of propagation (∼600 generations) to explore how the population-genetic dynamics of *P. aeruginosa* evolution differ in a model of the biofilm life cycle compared with a well-mixed environment (Flynn et al. 2016). This experiment was designed under biofilm-promoting conditions with a genotype already proficient in biofilm production, in contrast to previous studies (Lenski et al. 1991; Wong et al. 2012; Traverse et al. 2013; Turner et al. 2018). Major findings included that biofilm populations evolved greater phenotypic and ecological diversity than planktonic populations, and some of these phenotypes were linked to probable biofilm adaptations. The different colony types that evolved within populations represented distinct growth strategies that were most productive in mixture, suggesting a process of niche partitioning and facilitation among lineages (Flynn et al. 2016). The diversity among populations was also opposed to the phenotypic parallelism observed in previous studies (Rainey and Travisano 1998; Traverse et al. 2013). A preliminary survey of the genomes of these lineages suggested that this diversity was in part caused by the evolution of *mutS and mutL* genotypes that increased mutation rate, a genotype also observed in many chronic infections of *P. aeruginosa* (Ciofu et al. 2010; Macià et al. 2011; Warren et al. 2011; López-Causapé et al. 2013). Here, we use longitudinal whole-population genome sequencing to study the underlying evolutionary dynamics of this experiment at high resolution. Some genes that experienced mutational parallelism contribute to arginine metabolism, the sole carbon and nitrogen source in these experiments. More surprising, in light of the duration of the experiment and the strength of selection in these populations, few mutations actually fixed in any of the six populations and much more genetic diversity was preserved than has been seen in previous evolution experiments. We evaluate potential evolutionary and phenotypic causes for this maintenance of such levels of high genetic diversity.

## Results

### Experimental Evolution

Six populations of *P. aeruginosa* strain PA14, a strain already proficient in forming biofilm, were propagated for 90 days (∼600 generations, or ∼6.6 generations per day) to examine the population-genetic dynamics of prolonged growth in a biofilm life cycle. Bacteria that attach to a polystyrene bead are transferred daily to a new tube containing a fresh bead (populations B1, B2, B3) and compared with passages of serial 1:100 dilutions (planktonic populations P1, P2, P3) as described previously (Poltak and Cooper 2011; Flynn et al. 2016) (supplementary fig. S1, Supplementary Material online). Biofilm and planktonic populations were designed to ensure a similar transfer size and number of generations per day, as reported previously (Flynn et al. 2016). The culture medium was M63 media containing arginine as the sole carbon and nitrogen source and supplemented with 25 µM iron (see Materials and Methods), a combination which has been shown to promote biofilm production in *Pseudomonas* species (Bernier et al. 2011). We hypothesized that this media may select different adaptive mutations than those described in prior evolution experiments with *P. aeruginosa* (Barrett et al. 2005; Wong et al. 2012) because of its different metabolic demands and because biofilm production is externally induced, allowing us to identify subsequent steps of adaptation that are less known. We sought to study these population wide adaptations at both phenotypic and genetic levels with new experimental measures and whole-genome sequencing of both populations and clones.

**Fig. 1.**
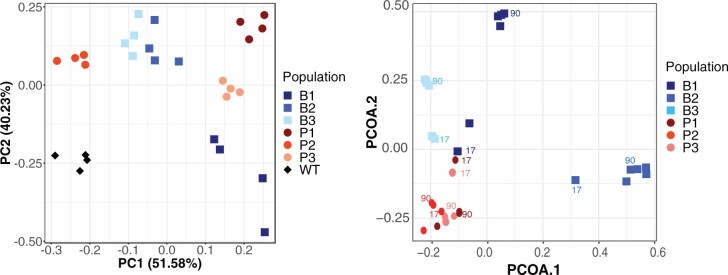
Divergent phenotypes linked to fitness and genotype frequencies distinguish each evolved population after 90 days of passage. (*A*) PCA of evolved phenotypes including biofilm production, maximum growth rate (Vmax), and swimming motility (*n* = 4 for each population, with each replicate being the average of one experiment block, see Materials and Methods for more detail). Blue squares are biofilm populations, red circles are planktonic populations, and the ancestor is indicated by black diamond(s). The first three components explain 51.58%, 40.23%, and 8.19% of variance. (*B*) Principal coordinate analysis (PCoA) of mutation identities and frequencies at days 17, 25, 44, 66, 75, and 90. Points from days 17 and 90 are labeled and colored by population to indicate trajectories. PCoA1 and PCoA2 explain 15.6% and 13.4% of the variance, respectively.

After 90 days, all six populations became vastly more fit than ancestral PA14, with selective coefficients 10-fold greater (*r* between 2 and 10, implying antagonism or killing of the ancestor in some cases; see Materials and Methods for details) than the fitness gains observed in other evolution experiments of similar duration (Lenski et al. 1991; Rainey and Travisano 1998; Barrett et al. 2005; Ellis et al. 2012; Wong et al. 2012) (supplementary fig. S2 and table S1, Supplementary Material online; see Materials and Methods). Five populations increased their maximum growth rate (Vmax; supplementary fig. S3*a*, Supplementary Material online) and five populations evolved to become less motile than the ancestor (supplementary fig. S3*b*, Supplementary Material online). Despite selection to attach to a plastic bead each day, only the B3 population evolved higher biofilm production than the ancestor as measured by the standard crystal violet assay. In contrast, four populations, including bead populations B1 and B2, evolved lower biofilm production (supplementary fig. S3*c*, Supplementary Material online). This result may indicate that the high starting biofilm production of PA14 in these conditions was difficult to improve. Alternatively, the lower biomass at 4 h may indicate that slower attachment is beneficial in the system; that is, higher biomass may cause biofilms to detach from the beads prematurely. We performed a principal component analysis (PCA) on these three phenotypes (fig. 1*a*) and found that evolved populations were distinguished from the ancestor but not clearly separated by treatment.

### High Genetic Diversity Arose and Persisted in All Populations

We used longitudinal whole-population genome sequencing to characterize the genetic diversity and evolutionary dynamics of each lineage with an average depth of 617.1 ± 142.3 reads per sample. A total of 145.7 ± 55 mutations were detected per population over the course of the 90-day time period (see Materials and Methods for filtering criteria). Biofilm populations accumulated more mutations (535, with 382 found at day 90) over the 90 days than planktonic populations (339, with 201 found at day 90; table 1). This number of mutations is remarkable in comparison to previous evolution experiments. For example, populations of *Burkholderia cenocepacia* (another opportunistic pathogen known for functional plasticity) were propagated using the same bead model, for twice the transfer days, and 37 mutations were identified in the best-studied replicate (Traverse et al. 2013). Biofilm populations were sampled at two more time points than planktonic to better capture their complex dynamics, discussed more below.

**Table 1.** Summary Statistics for Mutations Found in All Six Populations Following 90 Days of Experimental Evolution.

Total cumulative mutations	874
Biofilm only	535
Planktonic only	339
Total mutations at day 90	583
Biofilm only	382
Planktonic only	201
Total fixed mutations	53
Biofilm only	48
Planktonic only	5
Total fixation events	10

A phenomenon associated with positive selection is an increase in nonsynonymous (NS) mutations over synonymous (S) mutations (McDonald and Kreitman 1991; Kryazhimskiy and Plotkin 2008; Lieberman et al. 2011). In following with this trend, the normalized NS/S ratio for planktonic populations averaged 1.73 and for biofilm populations 2.34. A total of 31 indels were also identified in the six evolved populations (supplementary tables S2 and S3, Supplementary Material online); whereas, interestingly, no large structural variants were detected. A large number of intergenic mutations were also identified (233 mutations, or 27.5%), many in likely promoter or terminator regions. The importance of intergenic mutations still remains unclear; however, recent work has implicated intergenic regulatory regions as critical in *P. aeruginosa* evolution in the laboratory and during infections (Feliziani et al. 2014; Thorpe et al. 2017; Khademi et al. 2019).

We hypothesized that the structured biofilm environment would maintain greater genetic diversity than mixed liquid culture. This ecological process could potentially be at odds with effects of selection on beneficial mutations that would decrease genetic diversity. For example, a mutation that generally improves growth would be expected to sweep and eliminate genetic variation, despite biofilm growth being more likely to preserve coexisting subpopulations (Habets et al. 2006; Traverse et al. 2013; Martin et al. 2016). Planktonic populations would also be subject to similar effects of selective sweeps but their admixture might limit the cooccurrence of contending mutations (Lang et al. 2013). Consequently, comparing populations at a given time point might not adequately capture the longitudinal ecological and evolutionary dynamics affecting diversity. For this reason, we sampled biofilm populations at six time points (17, 25, 44, 66, 75, and 90 days) and planktonic populations at four time points (17, 44, 66, and 90 days) and focused on temporal changes in all mutations supported by three reads on each of both strands.

The nucleotide diversity of biofilm populations tended to be greater at the end of the experiment than after 17 transfers (day 17 vs. day 90: *P* = 0.0478, *t* = 2.821, df = 4 via two-tailed *t*-test). Planktonic populations continued to select for new mutations throughout the experiment, but they did not become more diverse by day 90 than at day 17 (*P* = 0.239, *t* = 1.384, df = 4 via two-tailed *t*-test), indicating a process where new mutations were displacing older ones. Biofilm populations also tended toward greater diversity than planktonic populations later in the experiment, albeit not significantly (planktonic vs. biofilm, day 90, *P* = 0.0749, *t* = 2.394, df = 4 via two-tailed *t*-test; supplementary fig. S4*a*, Supplementary Material online). These findings are consistent with the greater morphological variation in biofilm populations than planktonic ones that we reported previously (Flynn et al. 2016).

**Fig. 2.**
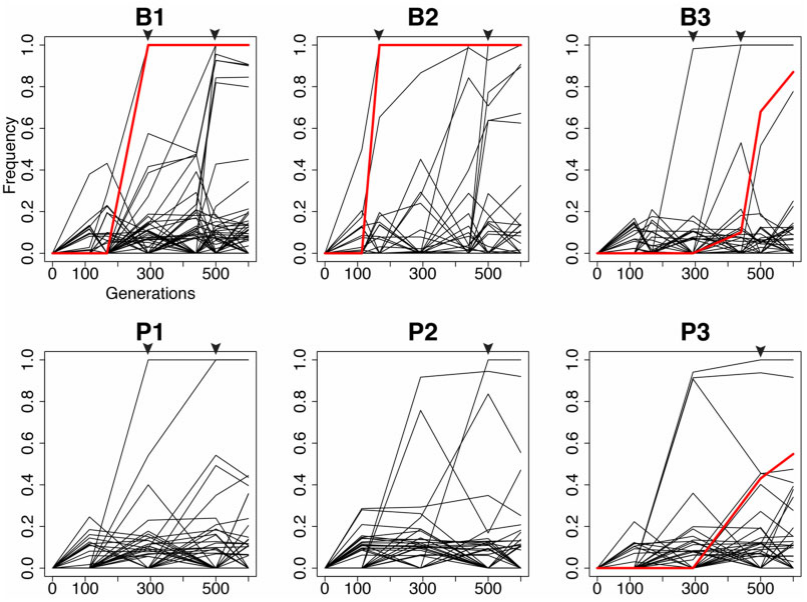
Evolutionary trajectories of inferred clonal lineages within six PA populations under biofilm (B) or planktonic (P) selection. Genotypes containing mutator alleles are colored red. Triangles indicate timing of the ten fixation events observed throughout the study.

A major cause of the high genetic diversity is the prevalence of mutator alleles, mutations that cause a genome-wide increase in mutation rate, in four populations. In all biofilm populations and one planktonic population (P3), NS mutations in the DNA mismatch repair (MMR) genes *mutS and mutL* became frequent or fixed. Two populations, B1 and B2, independently evolved the same *mutS* mutation (T112P) that has been shown to lower affinity for heteroduplex DNA in *Escherichia coli* (Junop et al. 2003) and results in a 116-fold increase in mutation rate in isogenic mutants (supplementary fig. S5, Supplementary Material online). This T112P mutation occurs within a homopolymeric region that may be a mutational hotspot. Genotypes containing these mutations fix in populations B1 and B2 by days 44 and 25, respectively (fig. 2, red trajectories). A premature stop mutation (W307*) in *mutS* also rose to intermediate frequency (53%) in the P3 population by day 90. Although we did not create an isogenic mutant of this mutator allele, we predict that it acts as other *mutS* mutations with a roughly 100-fold increase in mutation rate. The rise of *mutS* alleles caused these populations to diverge genetically from the others, as seen from principal coordinate analysis of mutational frequency data, which also distinguished biofilm from planktonic populations (fig. 1*B*). A fourth mutator genotype evolved in the B3 population by a mutation in *mutL* (D467G) that caused a 16-fold increase in mutation rate and rose to 83% frequency by day 90 (fig. 2; supplementary fig. S5, Supplementary Material online). Consequently, each of these mutator populations became enriched for transition and single-base indel mutations, signatures typical of defects in mismatch repair (Sniegowski et al. 1997; Shaver et al. 2002; Couce et al. 2017) (supplementary table S2, Supplementary Material online).

### Lineage Dynamics Demonstrate Ecological Differentiation and Clonal Interference

A more beneficial genotype, either containing one comparatively better mutation or having acquired several beneficial mutations whose combined fitness is superior, may be able to outcompete less fit lineages. However, the rate of increase of the fitter genotype can be slowed by this lineage competition, a dynamic is known as clonal interference or more generally as the Hill–Robertson effect (Birky and Walsh 1988; McVean and Charlesworth 2000; Comeron et al. 2008). Although mutator alleles are not beneficial themselves (Sniegowski et al. 1997) and their dominant effect is to introduce neutral or deleterious mutations, they can facilitate the acquisition of combinations of beneficial mutations. The ability to acquire many beneficial mutations and escape clonal interference may be particularly advantageous when beneficial genetic variation is rare or, more likely in these populations, common (Smith and Haigh 1974; Buskirk et al. 2017). This scenario predicts that invading mutator genotypes would comprise larger cohorts of putatively beneficial mutations than other contending lineages.

As described above, hundreds of mutations rose to detectable frequencies in these populations within ∼200 generations and persisted. We used software recently developed by our lab (Scribner et al. 2020) to group mutations into genotypes on the basis of shared, nested frequency-trajectories (Cooper et al. 2020; Deitrick 2020). Remarkably, between 30 and 47 genotypes, each composed of multiple mutations, rose to appreciable frequencies within each population (fig. 2). The number of genotypes found at a given sampled timepoint continuously increased in biofilm populations, whereas the maximum diversity in planktonic populations occurred at day 44 (supplementary fig. S4, Supplementary Material online). Yet despite extensive variation at both nucleotide and genotype levels, only ten fixation events involving a total of 53 mutations were observed. These selective sweeps involved genotypes with more mutations in biofilm populations than in planktonic lines (table 1), and two sweeps were linked to mutator alleles in which 24 mutations fixed (supplementary table S3, Supplementary Material online). These results suggest that the biofilm environment led to the fixation of larger mutational cohorts and more differentiated genotypes, whereas planktonic conditions selected smaller cohorts with one or few mutations to fix. These findings indicate that the rapid rise of mutator alleles coincided with multiple linked mutations and these more complex genotypes prevailed, especially in biofilms. It is possible that a single beneficial mutation linked to *mutS* or *mutL* genotypes caused the success of these genotypes, but the sheer number of different, fixed mutations, the dominant role of selection in these large populations, and the large degree of gene level parallelism (discussed further below) make this explanation unlikely.

**Fig. 3.**
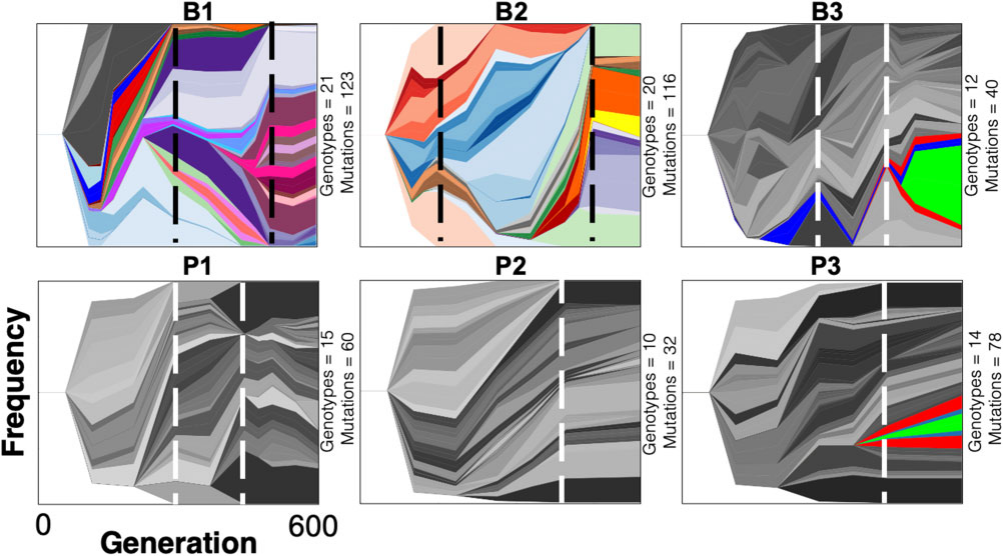
Genealogy and genotype frequencies over time. Each shade or color represents a different genotype and vertical area corresponds to genotype frequency, inferred by LOLIPop (Deitrick 2020). Shades of gray indicate genotypes not on a mutator background, whereas colors indicate genotypes on a mutator background. Dashed lines indicate timing of fixation events predicted by genotype trajectories (fig. 2). Biofilm populations are on the top row and planktonic populations are on the bottom row. The number of both genotypes and mutations present in each population at day 90 are indicated to the right of each panel.

To better understand the evolutionary processes within these populations, we visualized genotype frequencies over time by constructing Muller plots (Lang et al. 2013; Traverse et al. 2013). These figures demonstrate, for example, how one genotype spreads by evolving secondary, nested genotypes and outcompeting preexisting genotypes. The most conspicuous invasions were the mutator genotypes in populations B1 and B2 containing 11 or 13 mutations (fig. 3). These figures also illustrate effects of more consequential mutations that overtake others or give rise to further diversity as well as genotypes that arise simultaneously and coexist for hundreds of generations. This latter dynamic is consistent with either clonal interference or genotypes adapting to inhabit discrete niches, possibilities we evaluate below.

**Fig. 4.**
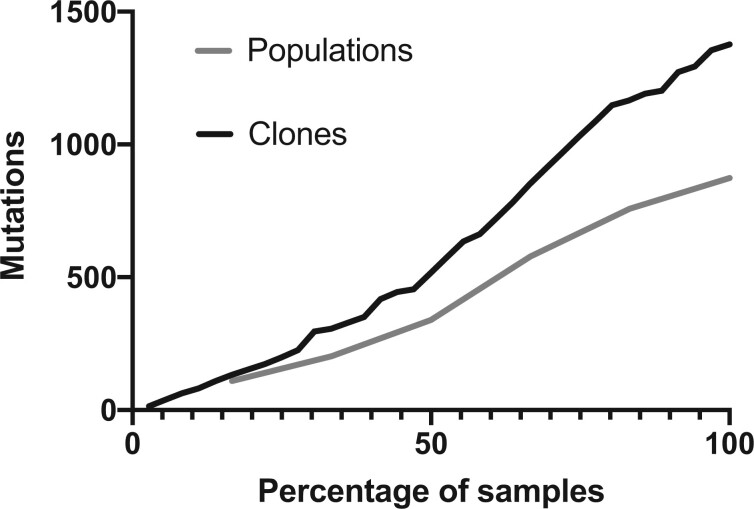
Sequencing clones identifies more genetic variation than whole-population sequencing. Collector’s curve of total mutations plotted by the percentage of samples (6 populations or 36 clones).

To test the genotype prediction algorithm producing these Muller plots and to identify other mutations undetected due to their low frequencies or sequencing coverage, we sequenced 26 clones from population B1 and 10 from population P1. B1 clones contained 48–138 mutations per clone whereas clones from the P1 population only contained between 14–28 mutations, results consistent with population sequencing results. However, many of these mutations were previously undetected in the metagenomes, indicating much greater genetic variation at frequencies below our detection limits from population sequencing (fig. 4) (Good et al. 2017). The 26 B1 clones belonged to a total of nine competing genotypes at day 90 (supplementary fig. S6, Supplementary Material online), whereas the ten planktonic clones only belonged to two distinct genotypes (supplementary fig. S7, Supplementary Material online). This finding confirms that the biofilm populations maintained greater genetic diversity than the planktonic populations at the end of the experiment.

**Fig. 5.**
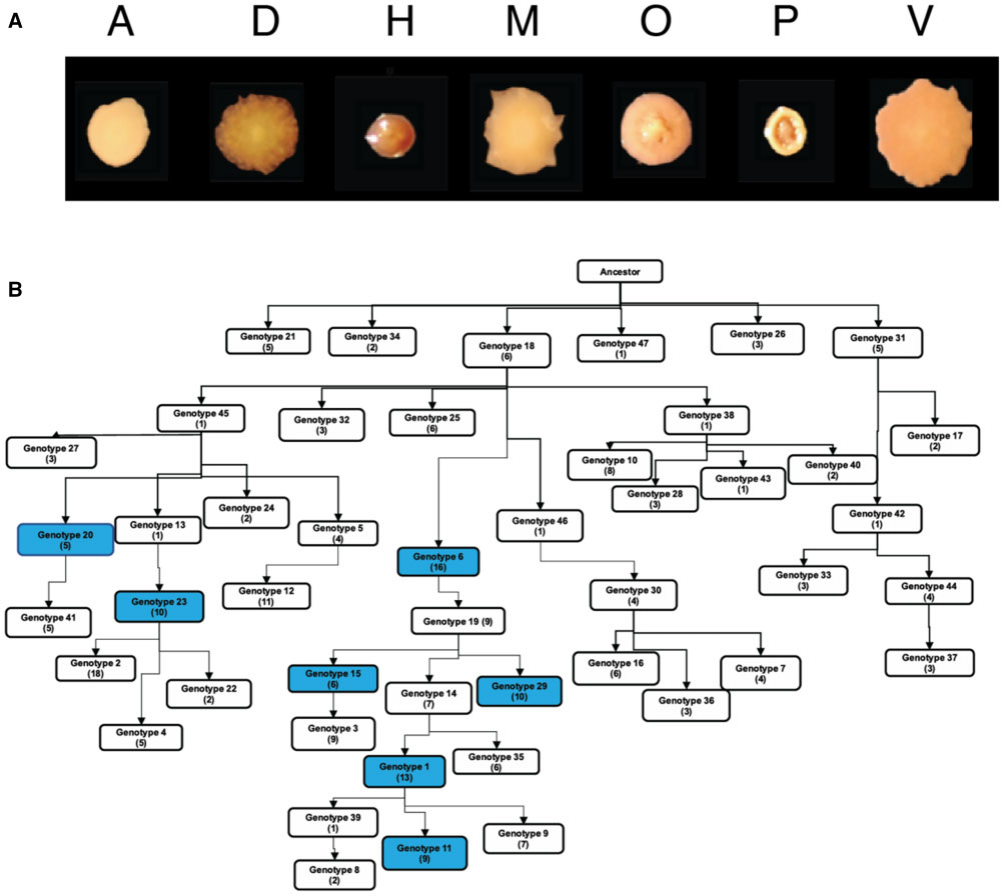
Clones representing different colony morphologies from the B1 population represent distinct lineages within the B1 population. (*A*) Seven colony morphologies were identified at day 90 and their phenotypes and ecological interactions characterized previously (Flynn et al. 2016). (*B*) Lineage ancestry was inferred using LOLIPop (Deitrick 2020). Genotypes of named morphotypes are shown in blue.

Another goal of sequencing these clones was to determine the genetic causes of the distinct colony morphologies that we previously showed to associate with distinct niches and phenotypes within the B1 biofilm population (Flynn et al. 2016). Each of the seven morphotypes (fig. 5*A*) represented a different long-lived genetic lineage (fig. 5*B*) that was separated by at least five mutations. This indicates that the previous study that was based solely on colony morphotypes was representative of the population diversity by including seven of nine identified lineages. We examined mutations unique to each genotype in an effort to identify those responsible for their unique phenotypes, which included the ability to form more productive biofilm communities in mixture (table 2).

**Table 2.** Unique Mutations Found in Seven Clones with Distinct Ecological Roles Isolated from an Experimentally Evolved Biofilm Populations (Flynn et al. 2016).

Clone	Gene	PA01 Ortholog	Pseudocap Function
A	*bkdA1*	*bdkA1*	
	*flgB*	*flgB*	Motility & Attachment
	*oprC*	*oprC*	Transport of small molecules
	PA14_06960	PA0534	Putative enzymes
	PA14_10830	No PA01 ortholog	Transcriptional regulators
	PA14_14530>/>PA14_14540		intergenic
	*gacS*	*gacS*	Two-component regulatory systems
	PA14_52760	PA0891	Transport of small molecules
	PA14_72320	PA5478	Membrane proteins
	*ppc*	*ppc*	Central intermediary metabolism
	*thrB*>/<PA14_72520		intergenic
	*trpB*	*trpB*	Amino acid biosynthesis and metabolism
D	*oprH*</>*napE*	*oprH*</>*napE*	intergenic
	PA14_04050	PA0227	Biosynthesis of cofactors, prosthetic groups, and carriers
	PA14_06030	PA0461	Fatty acid and phospholipid metabolism
	PA14_10910	PA4096	Transport of small molecules
	PA14_19450	PA3453	Hypothetical, unclassified, unknown
	PA14_23670	PA3127	Antibiotic resistance and susceptibility
	PA14_29260	PA2696	Transcriptional regulators
	PA14_31470	PA2557	Fatty acid and phospholipid metabolism
	PA14_48210	PA1239	Putative enzymes
	PA14_55380</<PA14_55390		intergenic
	*magD*	*magD*	Adaptation, protection
	PA14_66380	PA5021	Membrane proteins
	*pauR*	*pauR*	Carbon compound catabolism
	PA14_71750	PA5437	Transcriptional regulators
	*pvdJ*	*pvdJ*	Adaptation, protection
H	*algU*</>*nadB*	*algU*</>*nadB*	intergenic
	*dsbA2*	*dsbA2*	Translation, posttranslational modification, degradation
	*gbuR*	*gbuR*	Transcriptional regulators
	PA14_19190	PA3471	Central intermediary metabolism
	*prkA*	*prkA*	
	*fliT*	*fliT*	Motility & attachment
	*gacS*	*gacS*	Two-component regulatory systems
	PA14_58960	No PA01 ortholog	Hypothetical, unclassified, unknown
	PA14_71750	PA5437	Transcriptional regulators
	*rpoB*	*rpoB*	Transcription, RNA processing, and degradation
M	*flhA*	*flhA*	Motility & attachment
	PA14_07810	PA0599	Hypothetical, unclassified, unknown
	PA14_13150	PA3921	Transcriptional regulators
	PA14_43150</>PA14_43160		intergenic
	*pscQ*	*pscQ*	Protein secretion/export apparatus
O	PA14_12160	PA3992	Putative enzymes
	PA14_14390</>PA14_14400		intergenic
	PA14_27610>/>PA14_27620		intergenic
	PA14_27770	PA2812	Transport of small molecules
	PA14_51510	PA0988	Hypothetical, unclassified, unknown
	*pstA*	*pstA*	Transport of small molecules
	*spuF*	*spuF*	Transport of small molecules
	*str*	*str*	Antibiotic resistance and susceptibility
	*trbL*	*trbL*	
P	*lpxC*	*lpxC*	Cell wall/LPS/capsule
	*mdoH*	*mdoH*	Cell wall/LPS/capsule
	PA14_03000	PA0242	Putative enzymes
	PA14_13140>/>PA14_13150		intergenic
	PA14_13340>/<PA14_13350		intergenic
	PA14_18760	PA3523	Transport of small molecules
	PA14_43150	PA1652	Membrane proteins
	*pilF*	*pilF*	Motility & attachment
	*purB*	*purB*	Amino acid biosynthesis and metabolism
	*rep*	*rep*	DNA replication, recombination, modification, and repair
V	*rsmE*	*rsmE*	Translation, posttranslational modification, degradation
	*pqiB*	No PA01 ortholog	Hypothetical, unclassified, unknown
	PA14_52250	PA0929	Two-component regulatory systems
	*rsmI*	*rsmI*	Translation, posttranslational modification, degradation
	*thrB*>/<PA14_72520		intergenic
	*tli5a*>/>*pldA*		intergenic

For five morphotypes, we can predict a genetic cause of the high biofilm formation and the related high c-di-GMP levels. Morphotypes A and H each independently acquired mutations in *gacS*, a sensor kinase known to be associated with high biofilm production (Davies et al. 2007), and which will be discussed more below. The additional three evolved mutations in separate genes that have all been linked to *Pseudomonas* biofilm production previously: Mutant D acquired a mutation in the sensor histidine kinase *rcsC* (Nicastro et al. 2009), M acquired a mutation in the type III secretion system gene *pscQ* (Wagner et al. 2003), and V acquired a mutation in the two component response regulator PA14_52250 (Francis et al. 2017; Badal et al. 2020) (table 2). Although these are not the only mutations that distinguish the morphotypes from one another, these are leading candidates that could produce their varied biofilm phenotypes.

### Gene Targets of Selection

Evolving large populations (>10^8^) over a few hundred generations causes selection to dominate relative to the effect of drift, even when mutation rates increase. This allows us to predict that genotypes that rise to a detectable frequency are fitter than their ancestor. Further, parallel, nonsynonymous mutations affecting the same gene provides strong evidence of an adaptation (Bailey et al. 2017; Cooper 2018). Across all six populations, 53 mutations reach 100% frequency, as part of the ten genotype sweeps (table 1), and each affected a unique gene except for two each in *mutS*, *pitA*, and *rpoB* (supplementary table S3, Supplementary Material online). However, the population dynamics depicted in figure 3 and the clonal genomes summarized in figure 5 demonstrate substantial genetic variation that did not fix. Some genes were mutated in both treatments, indicating general adaptations to the growth conditions, whereas others were specific to lifestyle.

We analyzed the complete set of parallel mutations for those passing a statistical threshold of expected random co-occurrence and identified 153 genes or intergenic regions. Forty genes were mutated three or more times, more than would be expected by chance, accounting for 179 mutations (21.1% of all mutations; table 3). Most mutations are nonsynonymous, as expected from positive selection. The gene *rluB* (PA14_23110) acquired the most independent mutations, with ten mutations up to 17.7% frequency (table 3, supplementary table S3, Supplementary Material online). *RluB* encodes a 23s rRNA pseudouridine synthase responsible for modifying this rRNA at position 2605 during maturation (Kaczanowska and Rydén-Aulin 2007). As they are found in four populations, these are likely not lifestyle specific. However, all mutations clustered in residues 313 and 323 found in the disordered C-terminus of this protein. One additional mutation in *rluC*, another pseudouridine synthase, was detected. The literature remains vague about the specific phenotypic roles of these 23S rRNA modifications and effects of disrupting these genes are difficult to detect in vitro, but a recent study implicated these modifications as important for growth under anoxia (Krishnan and Flower 2008; Basta et al. 2017). Like *rluB/C*, many additional genes with parallel mutations are implicated in anoxia. Five mutations evolved in aerotaxis transducer *aer2*, which could produce an adaptive response to low oxygen that is common in biofilms (Sawai et al. 2012). Further, three independent V334G mutations were selected in PA14_46030, encoding the ortholog of biofilm dispersion locus BdlA (Morgan et al. 2006). This cytoplasmic protein contains two sensory PAS domains and a chemoreceptor domain that has been shown to sense and mediate responses to oxygen levels (Petrova and Sauer 2016). Although we do not know exactly how oxygen limitation influences fitness of these genotypes, these mutations allow us to predict that oxygen levels contributed to selection in these populations.

**Table 3.** Genes (*n* = 40) with Three or More Evolved Mutations.

Gene	Total Cases	Type			Biofilm	Planktonic	Highest Frequency						Function
		NS	S	Indel			B1	B2	B3	P1	P2	P3	
*argJ*	3	3	0	0	2	1		13.5	10.5			11.4	Arginine biosynthesis
*soxA*	3	2	1	0	1	2			10.6		14.7	11.4	Carbon compound catabolism
*aer2*	5	3	2	0	3	2		9.6	10.9	10.2		14.2	Chemotaxis
*bdlA*	3	3	0	0	3	0	14.4	14.7	13.5				Chemotaxis
*cobG*	3	2	0	1	1	2		61.8			13.6	13.1	Cofactor biosynthesis
*mutS*	3	3	0	0	2	1	100	100				52.6	DNA replication
*napF*	3	3	0	0	2	1	13.8		10.9			10.7	Energy metabolism
*nqrE*	3	3	0	0	1	2		11.7			10.4	11.2	Energy metabolism
*nuoG*	3	3	0	0	1	2	12.9			12.1	13.9		Energy metabolism
PA14_53110	7	7	0	0	2	5		14.8	12.8	12.9	14.4	12.1	Putative enzymes
*pslI*	7	7	0	0	3	4	16.7	16.4	14.6	16	14.7		Putative enzymes
*algF*	3	3	0	0	2	1	13.1			10.6			Secreted factors
*fha1*	4	4	0	0	3	1	27.6		25.1	35.8			Secreted factors
*rpoB*	4	4	0	0	4	0	100	66	100				Transcription
PA14_13150	3	3	0	0	3	0	46.3	17.8	100				Transcriptional regulators
PA14_18200	6	6	0	0	5	1	22.3	22.1		12.8			Transcriptional regulators
PA14_51840	3	3	0	0	0	3					12	16.6	Transcriptional regulators
PA14_58510	5	3	2	0	4	1	23	15.7		15.5			Transcriptional regulators
PA14_71750	3	3	0	0	3	0	93.9	89.7					Transcriptional regulators
*dsbA2*	4	2	2	0	3	1	17.1	11.4	20.8			10.5	Translation
*rluB*	10	8	2	0	4	6	10.8	17.7			14.7	13.2	Translation
*nosD*	5	4	1	0	1	4	14.1				15.3	12.9	Transporter
PA14_09300	3	3	0	0	2	1	16		15.2	18.6			Transporter
PA14_22650	7	7	0	0	3	4	43.2	24		40	28	37.8	Transporter
PA14_45060	6	6	0	0	2	4	24.4	19.1		19.5	14.1	13.6	Transporter
PA14_46110	7	7	0	0	1	6			15.5	13.7	13.6	10.7	Transporter
PA14_47900	3	3	0	0	0	3						19.9	Transporter
*pchF*	3	3	0	0	3	0	18.4		19				Transporter
*pitA*	4	2	0	2	2	3		91.1		100	75.8	100	Transporter
*pntB*	5	3	2	0	1	4	11.3				13.9	15.6	Transporter
PA14_10770	3	2	1	0	1	2		100		12.2		26.3	Two-component regulatory systems
PA14_32300	3	3	0	0	2	1	17.1					15.3	Two-component regulatory systems
*gacS*	3	3	0	0	3	0	85.2	92.2					Two-component regulatory systems
PA14_01160	3	3	0	0	1	2	14.3				13.8		Unknown
PA14_20510	6	6	0	0	3	3	9.6	11.5		11	11.9	11.1	Unknown
PA14_31070	10	5	5	0	4	6	19.3	16.9		16.2	16.9		Unknown
PA14_32830	6	6	0	0	2	4		13.8	12.7	12.1	13.5	12.1	Unknown
PA14_54810	3	3	0	0	3	0	11.7		7.5				Unknown
PA14_58070	6	6	0	0	5	1	13.6	17.5	12.9		20.8		Unknown
PA14_69010	5	5	0	0	3	2	20.1	12.2	13	12.5		12.4	Unknown

Note.—NS, nonsynonymous; S, synonymous; indel, insertion/deletion. Function from PseudoCap via pseudomonas.com.

Fortunately, a wealth of literature has identified genes involved in the transition from planktonic to biofilm growth in *P. aeruginosa* (Boles et al. 2004; Eichner et al. 2014). In addition to anoxia, two-component regulatory systems and mutations affecting motility are commonly implicated in this lifestyle transition. In agreement with this model, 19 mutations in 14 genes encoding sensor kinases, response regulators, or hybrid complexes were identified (table 3, supplementary table S3, Supplementary Material online). Further, nine different nonsynonymous or indel mutations affecting motility and attachment (e.g., *flgB/G, fliC/H/M, pilB/pilQ*, and *cupB3*) were selected in both biofilm and planktonic populations, indicating advantages of disrupting these processes in both lifestyles.

More surprising, the most common functional classification of mutated genes was transporters. Nine transporters were mutated repeatedly including: *pitA, pntB, nosD, pchF*, PA14_09300, PA14_22650, PA14_45060, PA14_46110, and PA14_47900, and account for 24% (43) cases of parallelism (table 3, supplementary table S3, Supplementary Material online). Of all transporters identified, *pitA* was the only one to reach frequencies above 50% in any population. This led us to identify an additional stressor acting on these populations as a byproduct of growth on arginine as a sole carbon source: an alkaline pH (pH = 9.2 at 24 h) caused by the release of ammonia through deamination (Ha et al. 2014). It remains unclear how these mutated transporters produce adaptations, but they could be acting to preserve phosphate under alkaline conditions that affect proton motive force (Harris et al. 2001). In addition to transporters, 18 mutations affected genes involved in various metabolic processes, including *argJ*, a key component of arginine biosynthesis expected to be no longer required due to abundant arginine in the growth media, *soxA*, encoding sarcosine oxidase (Willsey and Wargo 2016), and *cobG*, involved in the aerobic pathway of cobalamin (Vitamin B12) biosynthesis (Blanche et al. 1992). Finally, three mutations are observed in each of *napF*, *nqrE*, and *nuoG* potentially altering the energy conserving respiratory NADH dehydrogenase chain (Hoboth et al. 2009). We suspect that many of the nonsynonymous or indel mutations produced partial losses of function in metabolic pathways that are extraneous in the M63+arginine medium, though an exact mechanism is unknown.

There are four notable cases of mutated genes that are lifestyle specific. Four genes (*rpoB, gacS*, PA14_71750, and PA14_13150) are only mutated in biofilm populations, and in all cases, mutations rise to greater than 90% in at least one population (table 3, supplementary table S3, Supplementary Material online). Mutations in *rpoB* and other RNA polymerase genes (*rpoACD*) have been frequently identified as adaptations during evolution experiments (O’Sullivan et al. 2005; Xiao et al. 2017). One explanation in *E. coli* suggests that they are adaptive for growth in minimal media by redistributing RNA polymerase from small RNA promoters (i.e., rRNA) to rate-limited promoters required for anabolic processes from a limited set of carbon sources, such as arginine in the case of this study (Conrad et al. 2010). GacS is a sensor/regulator hybrid known to govern a broad range of traits involved in virulence, secondary metabolism and biofilm formation through the regulation of small rRNAs (Gellatly et al. 2018). Further, *gacS* has been shown to be involved in the switch from a hyperadherent small colony phenotype back to a wild-type phenotype indicating that loss of GacS function is beneficial in biofilms (Davies et al. 2007). The additional two biofilm specific loci, PA14_13150 and PA14_71750, are less understood transcriptional regulators; however, PA14_71750 has been previously associated with biofilm adaptation in *Pseudomonas* (Konikkat et al. 2021).

The rarity of fixation events and the high incidence of parallel mutations that likely produce similar phenotypes are consistent with competition between adaptive lineages, or clonal interference (Lang et al. 2013). Given a set of genes in which mutations are adaptive, we would expect each competing genotype to contain different combinations of these mutations. The most beneficial mutations would be seen repeatedly evolving on different genetic backgrounds. We identified 55 cases of within-population gene level parallelism (4–16 cases per population), with most (87%) predicted to be on different genotypes. These results support both clonal interference and incomplete sweeps of a trait comprised by different alleles, also known as a soft sweep (Hermisson and Pennings 2005; Lee and Marx 2013). The large number of mutated genes also demonstrates the polygenic capacity for adaptation by *P. aeruginosa* growing in minimal arginine medium.

### Selected Genotypes Are Adapted to Both Growth Conditions and Lifestyle

**Fig. 6.**
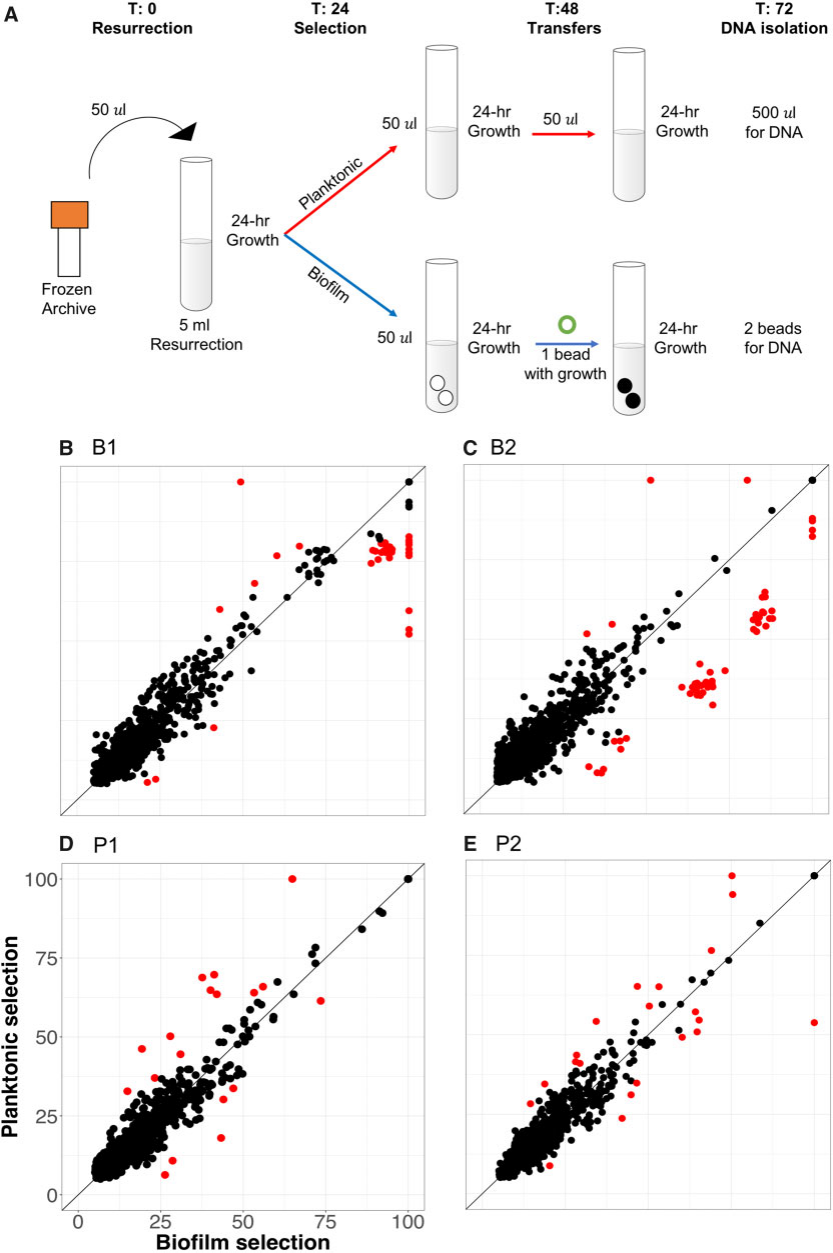
Effects of planktonic and biofilm selection on mutation frequencies within evolved populations. (*A*) Experimental approach. Aliquots of 90-day populations were resurrected under planktonic growth conditions for 24 h. Each population was then split and subjected to 2 days of either planktonic selection or biofilm selection on the bead model. After the second day of selection, genomic DNA was isolated from suspended or bound cells, respectively. (*B*–*E*) Correlation of mutation frequencies from planktonic or biofilm enriched treatments: (*B*) B1 population, (*C*) B2 population, (*D*) P1 population, and (*E*) P2 population. Mutation frequencies that depart significantly from expectations via Cook’s distance are represented in red and other mutations in black.

Most cases of gene-level parallelism were shared between biofilm and planktonic conditions and indicate adaptations to the common growth media. However, minority variants could include environment specific adaptations. For example, our bead transfer model simulates the complete life cycle of the biofilm—attachment, assembly, dispersal, and reattachment—each of which could select for discrete phenotypes. To distinguish genotypes adapted to either biofilm or planktonic growth, we grew 90-day samples of evolved populations B1, B2, P1, and P2 for 2 days in both of these conditions independently (fig. 6). We hypothesized that these treatments would enrich genotypes adapted to either condition that we could resolve by resequencing treated populations and correlating their relative frequencies. Mutation frequencies that are higher in one environment could represent genotypes that are adapted to biofilm or planktonic growth.

In all four populations tested, certain mutations were significantly enriched in both biofilm and planktonic environments (fig. 6*B*–*E*, supplementary table S4, Supplementary Material online). As expected, the two biofilm populations tested had more biofilm-enriched mutations than planktonic-enriched (37 and 55 biofilm-enriched compared with 4 and 2 planktonic-enriched), though planktonic populations had roughly equal numbers of mutations enriched in each condition (8 and 10 biofilm-enriched vs. 10 and 11 planktonic-enriched; supplementary table S4, Supplementary Material online). The B2 population contains a set of 36 mutations belonging to four genotypes enriched for biofilm fitness (supplementary table S4, Supplementary Material online), but these genotypes also rose in frequency between days 75 and 90 in the evolution experiment and may be generally adaptive. Overall, these experiments did not cause large (>10%) shifts in genotype frequency (supplementary table S4, Supplementary Material online), which suggests that the genotypes adapted to the overall experimental conditions as opposed to one of the specific lifestyle phases that we tested.

## Discussion

We used experimental evolution to examine how the opportunistic pathogen *P. aeruginosa* adapts in laboratory culture in a medium known to promote biofilm formation (Ha et al. 2014), comparing the results of propagation by simple serial dilution with a model simulating the biofilm life cycle (Poltak and Cooper 2011). After 90 transfers (∼600 generations) we observe many more mutations per population, fewer fixation events, and earlier prevalence of mutator genotypes than has been observed in other evolution experiments, including those in our own laboratory with a different opportunistic pathogen, *B. cenocepacia* (Traverse et al. 2013; Mhatre et al. 2020). These findings are consistent with two potentially overlapping processes: 1) the existence of many niches requiring different metabolism or growth strategies that select for varied specialized genotypes or 2) high clonal interference among genotypes of similar relative fitness in the same niche, which may lead to incomplete soft sweeps (Hermisson and Pennings 2005; Lee and Marx 2013). The repeated evolution of mutations in the same gene in the same population provides the clearest evidence of this clonal interference, because such mutants likely produce similar phenotypes. For example, within population B1, *gacS* mutations evolve independently in two predicted genotypes that we confirmed by clone sequencing (mutants H and A, table 2, supplementary table S3, Supplementary Material online). In our previous study, these clones were found to compete for a common biofilm-associated niche (Flynn et al. 2016). This indicates simultaneous adaptation to similar pressures in coexisting genotypes. In addition, we also predict that multiple niches exist within each population as our previous study indicated (Flynn et al. 2016). In that study, the increased productivity of the evolved population depended upon the presence of seven genotypes that differed in an array of biofilm phenotypes or niche dimensions. As further evidence, most cases of gene-level parallel mutations reached only 20–30% frequency within their respective populations (table 2). This finding is consistent with the evolution of niche specialists with different adaptations within each population, a process that has been shown in previous evolution studies, particularly with biofilms (Traverse et al. 2013; Frenkel et al. 2015; Buskirk et al. 2017; Good et al. 2017).

To identify the traits selected by growth in a minimal medium containing arginine as sole energy source, we focused on the 40 loci mutated most frequently (table 2). This medium was selected because it had previously been shown to induce high biofilm production by *P. aeruginosa* (Ha et al. 2014), specifically by increasing intracellular levels of the secondary messenger molecule c-di-GMP through the two diguanylate cyclase proteins RoeA and SadC. Additionally, arginine was the only amino acid to completely repress swarming motility, indicating this carbon source could be a cue to enter an attached lifestyle (Bernier et al. 2011). This design distinguished this experiment by promoting biofilm growth of the ancestor to enable study of subsequent adaptations. However, these conditions become stressful because arginine catabolism by arginine deiminase produces ammonia as a byproduct and produce a highly alkaline environment (Cunin et al. 1986; Lu 2006), which differ from previous evolution experiments with *P. aeruginosa* with glucose as a carbon source where the medium often becomes acidic (Bernier et al. 2011; Wong et al. 2012; Ha et al. 2014). The effects of arginine as the sole carbon and nitrogen source on the metabolic evolution of *P. aeruginosa* are complex and the topic of a future study; however, it is likely that this metabolism may have been a dominant pressure.

The remarkable, unexpected consequence of this experimental design was that selection did not act on mutations in a small number of shared genes in replicate populations. Rather, dozens or even hundreds of mutations in nearly as many genes appear to have been selected, suggesting a process of polygenic adaptation involving many metabolic and regulatory systems (fig. 3). These putatively adaptive mutations did not clearly depict distinct niches within the system, as prior studies have (Traverse et al. 2013; Frenkel et al. 2015). Such polygenic selection in large populations increases the probability of clonal interference, wherein each lineage acquires its own combination of adaptations, but any given genotype would struggle to become dominant. Consequently, fixation events were rare, with only ten events across all six replicate populations over 600 generations. Further, one of the most commonly mutated pathways was DNA mismatch repair, which increased mutation rates of linked genotypes in four populations. We hypothesize these mutator genotypes provided the means to escape clonal interference (Good et al. 2014) because they had a higher probability of producing multiple, linked beneficial mutations that could outcompete other genotypes, a phenomenon that has previously been associated with the introduction of alternative forms of structure (Raynes et al. 2019).

Our previous study of these populations predicted that the different colony morphotypes represented lineages that evolved to occupy distinct, interacting ecological niches within a biofilm community (Flynn et al. 2016), a model inspired by prior work with *Burkholderia* (Poltak and Cooper 2011). This genomic study confirms that these genotypes indeed represent different long-lived lineages within the B1 population, but each clone possessed several mutations that could explain their phenotypic variation, for example, fitness versus the ancestral PA14 strain, levels of cyclic-di-GMP, and motility (Flynn et al. 2016). Further, considerably more genetic variation was detected by population sequencing than these lineages could explain, and likewise, sequencing additional clones resulted in an ever-increasing census of new mutations (fig. 4). These results reinforce a polygenic model of adaptation to the biofilm-inducing arginine medium and the potential for niche differentiation within this environment (Flynn et al. 2016; Barghi et al. 2020).

More generally, this work has implications for understanding processes underpinning the evolution and maintenance of high levels of genetic diversity, including effects of different nutrient sources. Although the growth mode has been shown to influence genetic diversity (O’Toole et al. 2000; Boles et al. 2004; Resch et al. 2005; Booth et al. 2011), the metabolic environment may be equally important by exposing varied fractions of the genome to selection. Finally, this study demonstrates how microbial populations evolving in a new environment can adapt and diversify without the periodic losses of genetic variation caused by hard selective sweeps. This dynamic was demonstrated in the Long-Term Evolution Experiment with *E. coli* after many thousands of generations had passed and the rate of adaptation had slowed (Couce et al. 2017; Good et al. 2017). Yet here, populations amassed extreme fitness gains over only hundreds of generations while maintaining many lineages, suggesting that the *P. aeruginosa* genome encodes vast potential to meet new environmental challenges.

## Materials and Methods

### Experimental Evolution

Replicate populations were experimentally evolved as previously reported (Flynn et al. 2016). Briefly, the ancestral *P. aeruginosa* (PA14; NC_008463) strain was reconstituted from a freezer stock in Luria–Bertani broth (LB; 1.0% w/v tryptone, 0.5% w/v yeast extract, 1.0% w/v NaCl). Six replicate populations were started with 1:100 dilutions of an overnight growth into 5 ml M63 media (15 mM (NH_4_)_2_SO_4_, 22 mM KH_2_PO_4_, 40 mM K_2_HPO_4_, 40 mM galactose, 1 mM MgSO_4_, 25 μM FeCl_2_, and 0.4% w/v L-arginine [Bernier et al. 2011]). Three populations were propagated under liquid grown, planktonic, selection (P1, P2, P3), whereas three populations were propagated, concurrently, under constant biofilm selection (Poltak and Cooper 2011; Traverse et al. 2013; Flynn et al. 2016) (B1, B2, B3). Planktonic selection consisted of daily 1:100 liquid dilutions into 5 ml M63 media, whereas biofilm selection consisted of the transfer of one 7-mm polystyrene bead every 24 h to a new tube of 5 ml M63 media containing one clean bead. This biofilm selection method requires the entire biofilm life cycle of dispersal, attachment, and growth between every transfer. All six populations were propagated for 90 days (∼600 generations) in 18 × 150 mm test tubes at 37 °C on a roller drum at 30 rpm.

Archives were made for all populations at days 17, 25, 33, 45, 66, 75, and 90 in 8% dimethyl sulfoxide (DMSO) at −80 °C. Planktonic populations were archived by freezing a 1-ml aliquot of the 24-h culture, whereas biofilm populations were archived by sonicating 48 h beads in 1 ml phosphate-buffered saline (PBS), and then freezing the PBS with 8% DMSO.

### Phenotypic Characterization of Evolved Populations

All populations were revived from freezer stocks for characterization by adding 50 µl of freezer stock to a culture of 4:1 M63 media to LB media, with trace elements to recreate previous water mineral content (110.76 g/l CaCl_2_, 0.82 g/l MnSO_4_, 33.48 g/l KBr, 132.74 g/l Na_2_SiO_3_, 123.49 ZnSO_4_, 0.18 g/l CoCl_2_*6H_2_O, 1.46 g/l CuSO_4_). This media combination was used to minimize selection effects of growing in a fully complex medium, while providing enough nutrients for the frozen cells to revive. Resurrected populations were grown for 24 h at 37 °C on a roller drum before being vortexed for 10 s, twice, to ensure biofilm growth was disrupted from the sides of the glass tube. A total of four blocks, or for separate resurrections, were performed for the following three phenotypic assays. Data reported in supplementary figure S3, Supplementary Material online, are all raw data for all blocks, but figure 1*A* includes only the averages of each block.

Growth curves were measured for resurrected populations in 96-well plates, at 37 °C. All growth curves were started at an OD_600_ of 0.01 and growth was measured every 10 min following 9 min of shaking for 24 h in M63 media with trace elements. Maximum growth rate was determined for the average of all replicates, by using the following equation:

$$r=\frac{\Delta \;\text{log}({\mathrm{OD}}_{600})}{\Delta t}.$$

Census population size was calculated by diluting resurrected populations 1:100 in 5 ml M63 media with trace elements. One 7-mm polystyrene bead was added to each biofilm culture at time zero. After 24 h growth, all populations were vortexed for 30 s and then diluted and plated in triplicate on tryptic soy agar and grown at 37 °C for 48 h. Average colony counts of the three technical replicates were reported for each population. Additionally, populations plated for census population size were also used for pH measurements as they were complete replications of experimental conditions. Average pH of triplicate readings is reported.

Biofilm assays were performed on resurrected populations diluted to an OD_600_ of 0.01 in M63 media and grown in a 96-well plate under static conditions for 4 h at 37 °C. Media was discarded and the plate was washed twice with deionized H_2_O, which was also discarded. Wells were then stained with the addition of 250 µl 0.1% crystal violet solution for 15 min. Plates were rinsed to remove excess dye and the plate was allowed to dry for 24 h. A de-stain solution (95% ethanol, 4.95% dH_2_O, and 0.05% Triton X-100 [fisher bioreagents]) was added to wells (250 µl per well), and, after a 15-min incubation, was transferred to a new 96-well plate. Crystal violet absorbance readings were measured at 590 nm. Reported values are the average of 21 replicates, done across three plates (seven technical replicates per plate). Data were analyzed with a one-way ANOVA with post hoc Tukey test.

Motility assays were performed as described previously (Ha et al. 2014). Briefly, pipet tips were dipped into resurrected populations and stabbed into motility agar (0.3% agar, 6 g/l Na_2_HPO_4_, 3 g/l KH_2_PO_4_, 0.5 g/l NaCl, 0.2% glucose, 0.5% casamino acids, 1 mM MgSO_4_). Increased or decreased motility was determined for all day 90 evolved populations as the diameter of bacterial growth after 24 h growth at 37 °C compared with the clonal ancestor. Reported values are the average of five replicates. Results were analyzed by one-way ANOVA with post hoc Tukey test.

### Fitness Competitions

The fitness of 90-day evolved populations relative to the ancestor was measured in both planktonic and biofilm conditions. Competitors were revived from freezer stocks in 5 ml LB using 50 µl of freezer stock and incubating for 24 h. Equal volumes of each competitor were inoculated into 5 ml M63+arginine media for planktonic competitions, with the addition of two beads for biofilm conditions. All competitions were replicated six times. Samples were enumerated by plating on half-strength tryptic soy agar at 0 and 24 h, using a neutral *lacZ*-marked ancestor for differentiation. Fitness was calculated as the selective rate constant (*r*) using the following equation, where d = day:

$$r=\frac{\ln\frac{\mathrm{Evolve}d_{d=1}}{\mathrm{Evolve}d_{d=0}}-\ln\frac{\mathrm{Ancesto}r_{d=1}}{\mathrm{Ancesto}r_{d=0}}}{2}.$$

In some replicates, the ancestral competitor became nearly undetectable after 24 h, rendering fitness estimates unreliable, so these were omitted.

### Mutation Rate Estimate

Isogenic mutants of biofilm-evolved mutator alleles *mutS* T112P and *mutL* D467G were used as reported previously (Flynn et al. 2016). Briefly, the two mutator strains and the PA14 ancestor were revived from freezer stocks by streaking on ½ trypic soy agar. After 24 h growth individual colonies were used to start 30 replicate 5-ml cultures for each strain. After 24 h growth at 37°C on a roller drum, populations were diluted and plated on ½ tryptic soy agar both with and without 1 mg/ml ciprofloxacin as a selective agent. Colonies were counted after a 24-h incubation period on both the antibiotic and nonantibiotic plates. We then calculated the fold change in mutation rate using the maximum likelihood method of Gerrish (2008). We performed measurements for all strains simultaneously to minimize variation. Reported values are the average fold change of all replicates.

### Genomic Sequencing of Evolved Metagenomes

DNA was isolated from biofilm populations at 113, 167, 293, 440, 500, and 600 generations (17, 25, 44, 66, 75, and 90 days) and from planktonic populations at 113, 293, 500, and 600 generations (17, 44, 66, and 90 days). Culture media for DNA isolation was composed of 4:1 M63 to LB media. DNA was isolated from 1 ml resurrected populations using Qiagen’s DNeasy Blood & Tissue Kit. Library construction was done using the Illumina Nextera kit as described previously (Baym et al. 2015). Libraries were sequenced on Illumina’s NextSeq 500 platform by the Microbial Genome Sequencing Center at the University of Pittsburgh. Between 13,545,344 and 46,890,487 reads were obtained for each sample, resulting in coverage of 265–860× per sample.

### Determining Mutational Frequencies from Sequencing Data

Initial metagenomics sequencing efforts of the six populations resulted in 121.2 Gb of 2 × 151 bp sequencing reads that we trimmed and quality filtered with trimmomatic v0.36 using default parameters (Bolger et al. 2014). The breseq software package v0.31.0 was used to align the filtered reads to the reference PA14 genome (NCBI’s RefSeq database: NC_008463.1) and make polymorphism calls (Barrick et al. 2014). All populations were run using the polymorphism mode of breseq with a sliding window of 5 bp and a quality score cutoff of 10. This analysis was done for every population and every time point sequenced. The genome of the ancestral strain differed from the reference by 435 mutations, which were removed from all evolved population samples before any downstream analysis to remove mutations that did not evolve over the course of the evolution.

All 12,250 resulting mutation calls were filtered in R (v. 3.5.1)(R Core Team 2020) to require a mutation to be called by at least three reads on each strand (positive and negative). Finally, mutations were filtered to remove regions of high polymorphism. Due to the abundance of repetitive regions in the PA14 genome, the false positive rate is high. We therefore examined each call using the alignments produced by breseq and excluded mutations with high sequence variation within the 15 bases both upstream and downstream of the called mutation. Mutations were also filtered for known regions of misalignment such as repeat regions before consolidation into time-series tables containing all real mutated loci for each evolved population (supplementary table S1, Supplementary Material online). The resulting 874 mutations were then used as input in the software package LOLIPop Version 0.8.1 using –similarity-cutoff value of 0.1 for P1, P2, P3, and B1 and a –similarity-cutoff of 0.2 for populations B2 and B3. Increased similarity cutoffs increased the number of mutations that were grouped into a genotype. All scripts for the R filtering and analysis are available on github at: https://github.com/KatrinaHarris23/PALTEanalysis.

The neutral ratio of nonsynonymous to synonymous substitutions (d*N*/d*S* ratio) was estimated for the ancestral PA14 genome (http://www.kazusa.or.jp/codon/) as 2.96. All reported d*N*/d*S* ratios of mutated genes or genomes were standardized relative to this neutral expectation.

### Test of Environment-Specific Selection by Population Resequencing

To test whether mutations were selected specifically to the biofilm lifestyle steps of planktonic growth or surface attachment, we isolated DNA from evolved populations after additional strong selection in two distinct environments (fig. 5). Populations were resurrected from day 90 archives for the B1, B2, P1, and P2 populations by using 50 µl of the frozen stock into 5 ml LB. After 24 h growth at 37 °C, each culture was used to inoculate two 5 ml M63+arginine tubes. Two 7-mm polystyrene beads were added to one tube and both cultures were grown at 37 °C. Each population was transferred at 24 h, via serial dilution of the liquid phase for planktonic selection and by transferring the bead, following rinsing in PBS, to a fresh media with oppositely colored beads. After a second 24 h incubation at 37 °C, DNA from the planktonic treatment was isolated from 0.5 ml liquid phase, whereas DNA from the biofilm treatment was isolated from cells attached to the colored beads after a rinse in PBS, via sonication. The Qiagen DNeasy Blood and Tissue kit was used for both.

Between 328 and 410× coverage was obtained for all eight samples. Mutations were filtered by removing all ancestral mutations, and only mutations detected by both biofilm and planktonic samples for a given population were considered. This sequencing effort resulted in 979, 1021, 879, and 876 mutations in the B1, B2, P1, and P2 populations, respectively. Relative frequencies of mutations in the two environments were plotted in R using the ggplot2 package (supplementary fig. S8, Supplementary Material online; code used in this analysis is found on github https://github.com/KatrinaHarris23/PALTEanalysis/blob/master/201020_ecological_interactions.R).

### Clonal Sequencing

To acquire clonal DNA, populations were resurrected in 4 ml M63 arginine media with 1 ml LB and grown for 24 h at 37 on a roller drum. Populations were then plated on ½ Tsoy plates down to the −6 dilution to isolate individual colonies. After 24 h of growth at 37°C followed by 48 h at room temperature, clones were selected to sample all distinct colony morphologies. Colonies were picked and used as inoculum for 5 ml LB cultures. After 24 h at 37 on a roller drum, 1 ml of culture was used for archiving (9% DMSO) and 0.5 ml was used for DNA isolation. Named clones that were reported previously (Flynn et al. 2016) were already archived and inoculated directly into 5 ml LB.

DNA sequencing was performed as with population sequencing, but only requiring 30× coverage per sample. Variant calling followed methods described above except filtering only excluded ancestral mutations before analysis.

### Statistical Testing

Alpha diversity was calculated for all detected mutations in each population at each time point. We used the “shannon,” “simpson,” and “invsimpson,” modes of the diversity function in R. Populations were grouped by growth environment, biofilm (*n* = 3) and planktonic (*n* = 3), and by mutator allele presence (*n* = 4) or absence (*n* = 2). *T*-tests were performed in Prism (version 8, www.graphpad.com, supplementary fig. S4, Supplementary Material online).

To test whether parallelism was greater than expected by chance, a table of loci observed mutated two or more times was generated from the final mutation calls. Locus length was determined using http://pseudomonas.com as either gene length or the distance between surrounding genes for intergenic regions. Fisher’s exact test was performed using this compiled list: the numbers of mutations per loci, the total number of mutations observed (624), and the PA14 genome length (6,537,648). Testing was performed using the fisher.test() function in R and only loci with *P*-values lower than 0.05 were used for parallelism analysis. In total, 153 loci were tested for parallelism and then the number of false positives were reduced using the Benjamini–Hochberg correction with a false discovery rate of 5%. The equation used was (*i*/*m*)*Q* where *i* is the *P*-value rank, *m* is the total number of tests, and *Q* is the false discovery rate.

Finally, ecological enrichment significance was calculated in R by using Cook’s distance. First, a linear regression was calculated for each population using planktonic versus biofilm frequencies using the lm() function. Cook’s distance was then calculated for each point, using cooks.distance() function. A mutation was considered significantly enriched if its distance was four times the mean distance or more.

### Principal Component Analysis

All PCA calculations and plotting were performed in R. Principal components were computed from the biofilm production, swimming motility, and maximum growth rate (Vmax) data, and from the mutational frequency data at each sampled day using the prcomp() function. Plotting was done using the autoplot() function that is part of the ggfortify package.

## Supplementary Material

Supplementary data are available at *Molecular Biology and Evolution* online.

## Supplementary Material

msab248_Supplementary_Data
