# Supplementary material for: Polygenic Adaptation and Clonal Interference Enable Sustained Diversity in Experimental Pseudomonas aeruginosa Populations
Source: Mol Biol Evol. 2021 Aug 19;38(12):5359–75. doi: 10.1093/molbev/msab248 (PMC8662654; doi:10.1093/molbev/msab248)
Supplement: msab248_Supplementary_Data [file msab248_supplementary_data.pdf]

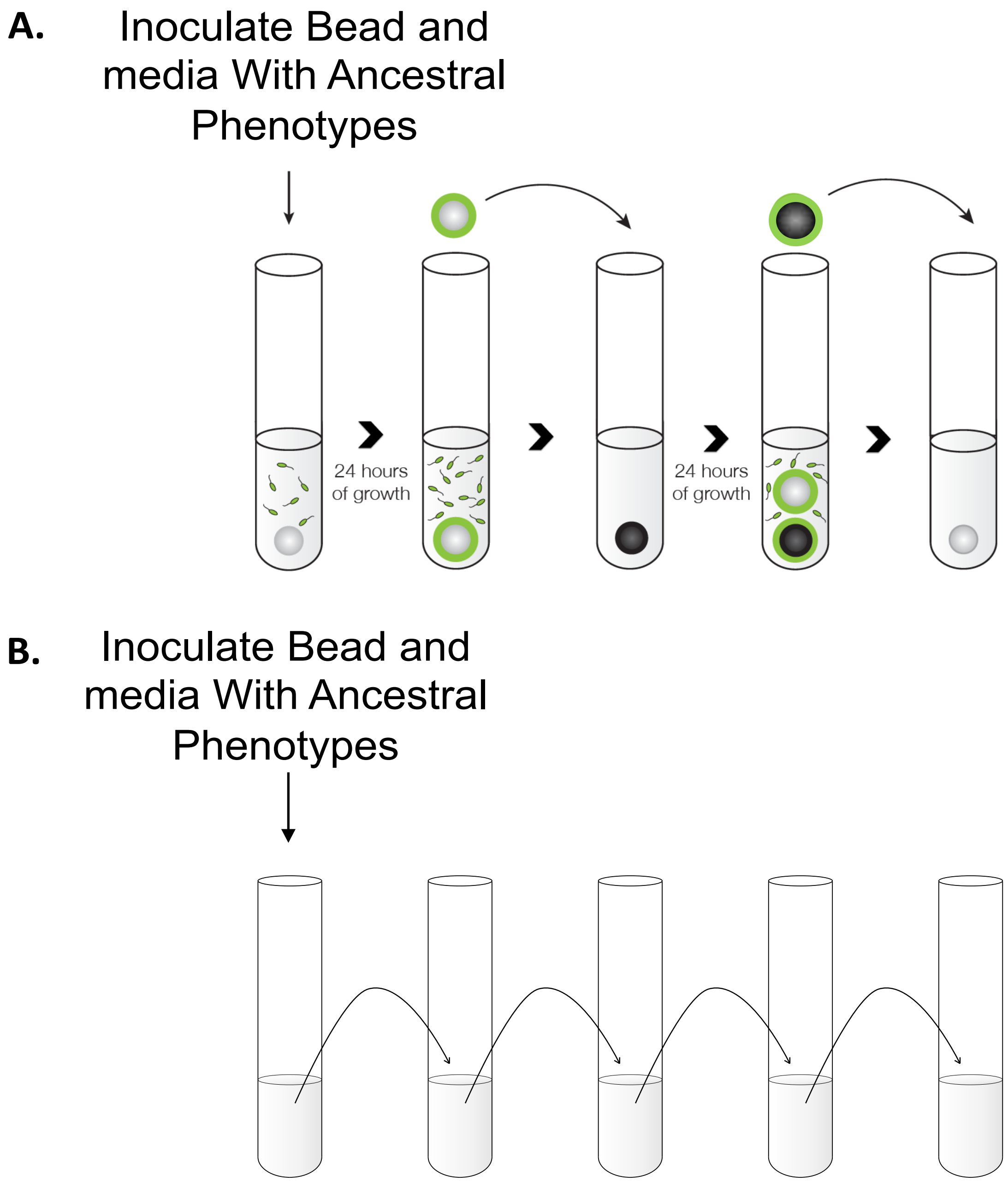

Figure S1. Schematic for *Pseudomonas aeruginosa* experimental evolution[10]. (A) Biofilm populations were propagated by transferring a colonized bead to a new tube with an uncolonized bead daily. (B) Planktonic populations were propagated in liquid media with 100-fold dilutions every 24 hours.

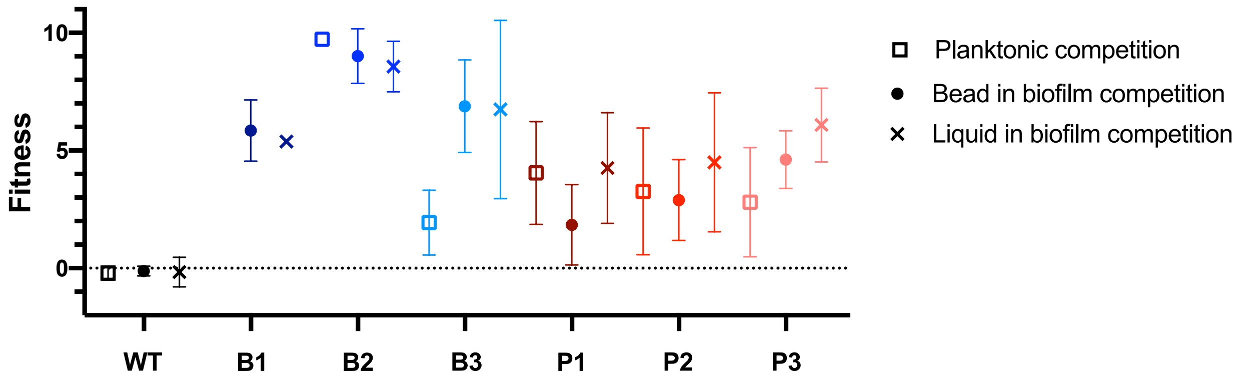

Figure S2. Relative fitness of evolved populations sampled after 90 days versus the PA14 ancestor, measured as selection rate constant *r*. Biofilm competitions were sampled from two growth phases: from cells attached to the bead (filled circles) and from the liquid growth phase (x). The B1 population outcompeted the ancestor in the planktonic environment to the point where the ancestor was undetected at 24 hours, so no result is shown. B2 populations also nearly excluded the ancestor.

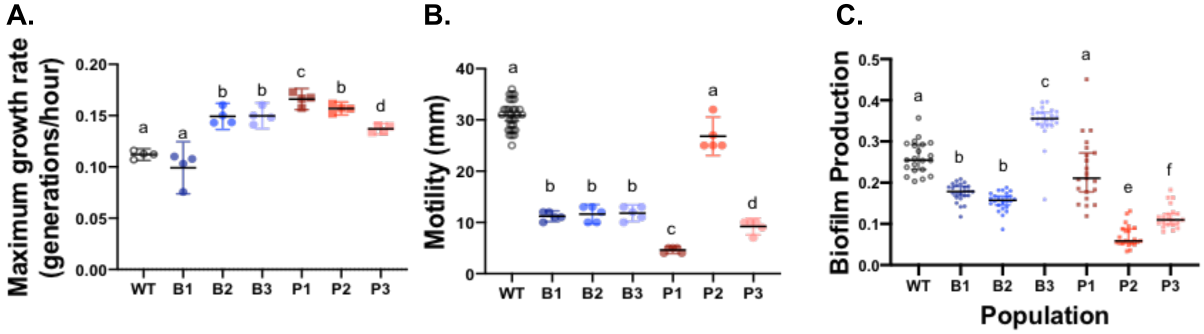

Figure S3. Traits tied to fitness diversified among replicate populations. 90 day evolved populations were tested for (A) maximum growth rate; n=4, (B) swimming motility; n = 5, (C) and 4-hr biofilm production; n = 21. All individual populations (B1 = dark blue circles, B2 = blue circles, B3 = light blue circles, P1 = dark red squares, P2 = red squares, P3 = orange squares) were compared against the ancestral, WT, strain (black open circles). All data points are indicated with symbols, with means and 95% CI represented with vertical bars. Letters indicate populations significantly different from one another. There were significant differences observed in all sampled characteristics (ANOVA with Tukey's post hoc testing for A) biofilm production: F=55.17, $p < 10^{-4}$, B) motility: F=189.40, $p < 10^{-4}$. C) maximum growth rate: F=36.15, $p < 10^{-4}$).

Table S1. Raw data for fitness calculations. Colony counts are colored by day with counted values under 30 in orange and values of 1 put in for 0's highlighted in yellow.
https://github.com/KatrinaHarris23/PALTEanalysis/blob/master/Table_S1.xlsx

	All	B1	B2	B3	P1	P2	P3
**Mutations:**	874	239	180	116	109	94	136
**Nucleotide level mutations**							
**Indels:**	31	13	10	2	2	1	3
**Transitions:**	246	107	86	8	6	2	37
**Transversions:**	595	118	84	105	101	91	96
**Amino acid level mutations**							
**Coding:**	27	11	8	3	2	1	2
**Intergenic:**	230	53	34	39	37	28	39
**Pseudogene:**	10	3	1	4	1	0	1
**Premature stop:**	9	3	0	2	1	1	2
**Elongating:**	11	4	3	1	2	0	1
**Synonymous:**	105	33	25	6	16	10	15
**Non Synonymous:**	478	131	108	60	50	53	76
**dN/dS:**	1.75	1.53	1.66	3.85	1.20	2.04	1.95

Table S2. Mutation statistics of all unique loci mutated in the six evolved populations. All unique loci mutated within each population at any time point sequenced are included. dN/dS ratios are standardized to the PA14 genome neutral ratio (see methods).

Table S3. All mutation calls determined through whole genome, whole population sequencing of the six populations of PA propagated for 90 days after filtering; see methods for filtering criteria.
https://github.com/KatrinaHarris23/PALTEanalysis/blob/master/Table_S3.csv

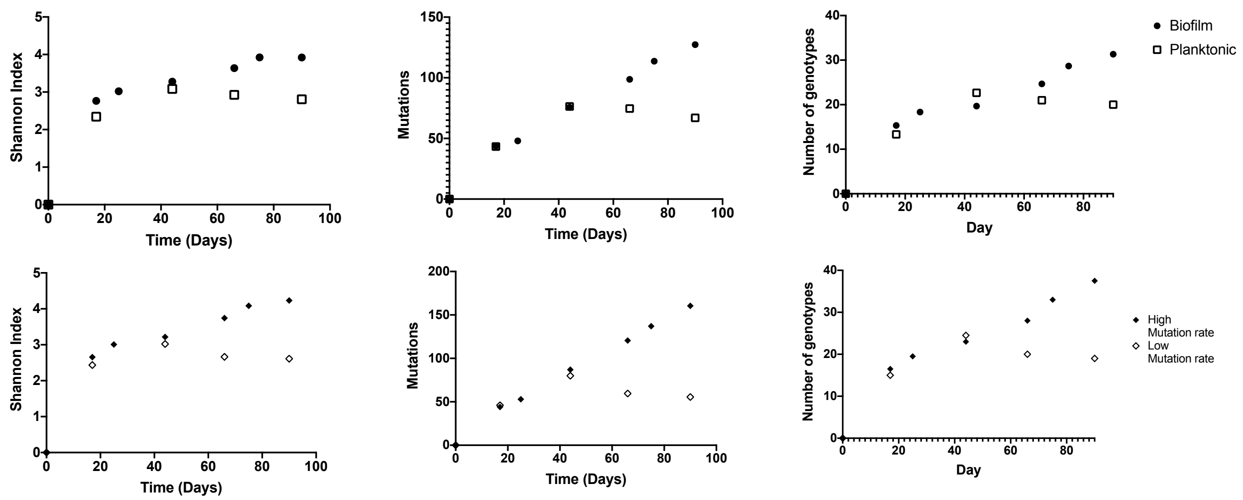

Figure S4. Biofilm populations become more diverse over time (filled circles in top row), in terms of alpha diversity (p-value = 0.0280), but the change was not significant in terms of individual mutations (p-value = 0.195) or predicted genotypes (p-value = 0.204) plotted through time. Planktonic environments (open squares in top row) do not significantly increase in any of these metrics (p values = 0.9261 for diversity, 0.9296 for number of mutations, and 0.5539 for number of genotypes). Similarly, when we compare high mutation rate (B1 and B2; filled diamonds in bottom row) to low mutation rate (P1 and P2; open diamonds in bottom row) populations we see a significant change in high mutation rate, despite the lack of power, in numbers of mutations (p-value = 0.0299) and number of genotypes (p-value = 0.0373), however diversity does not significantly change over the 90 days (p-value = 0.9523). P-values are from one way ANOVA’s corrected for multiple comparisons.

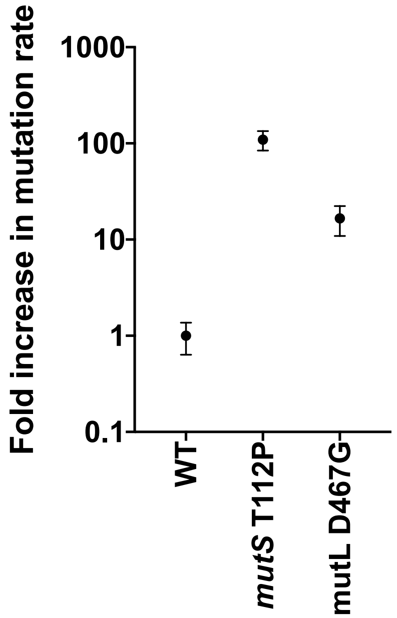

Figure S5. Mutation rate increase of *mutS* T112P and *mutL* D467G isogenic mutants. The two mutator alleles that evolved in biofilm environments result in a 109-fold and 16-fold increase in mutation rates over the ancestral strain.

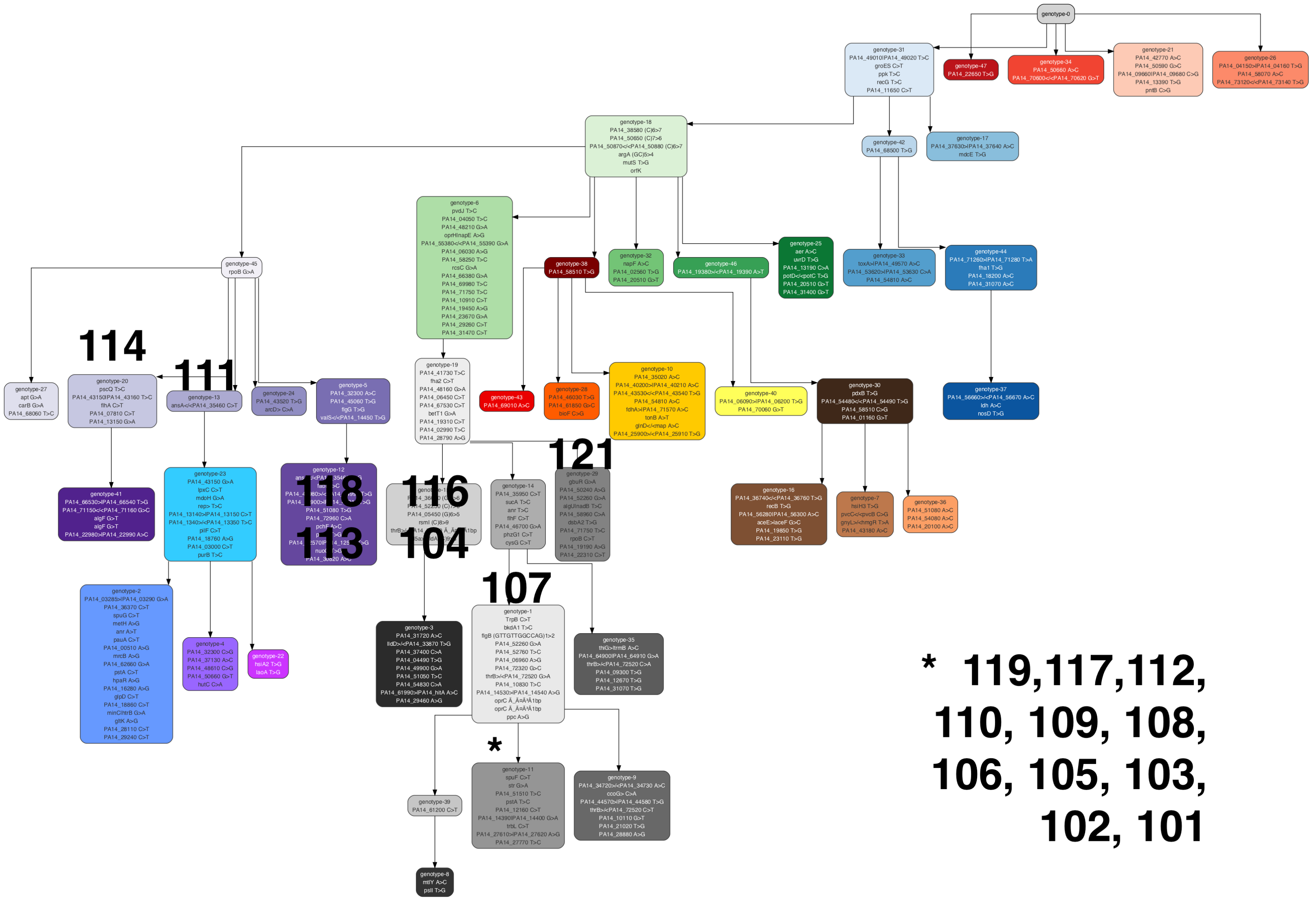

Figure S6. Genotype ancestry plot showing the ancestral relatedness of genotypes in population B1. Different colors indicate different genotypes, with shades of the same color indicating the same lineage, with time on the vertical axis.

Clones isolated from the B1 population belong to 7 nodes on the B1 population ancestry, indicated by numbers. Colors denote ancestral lineages determined by the LOLIPOP package and do not correspond to any previous figures.

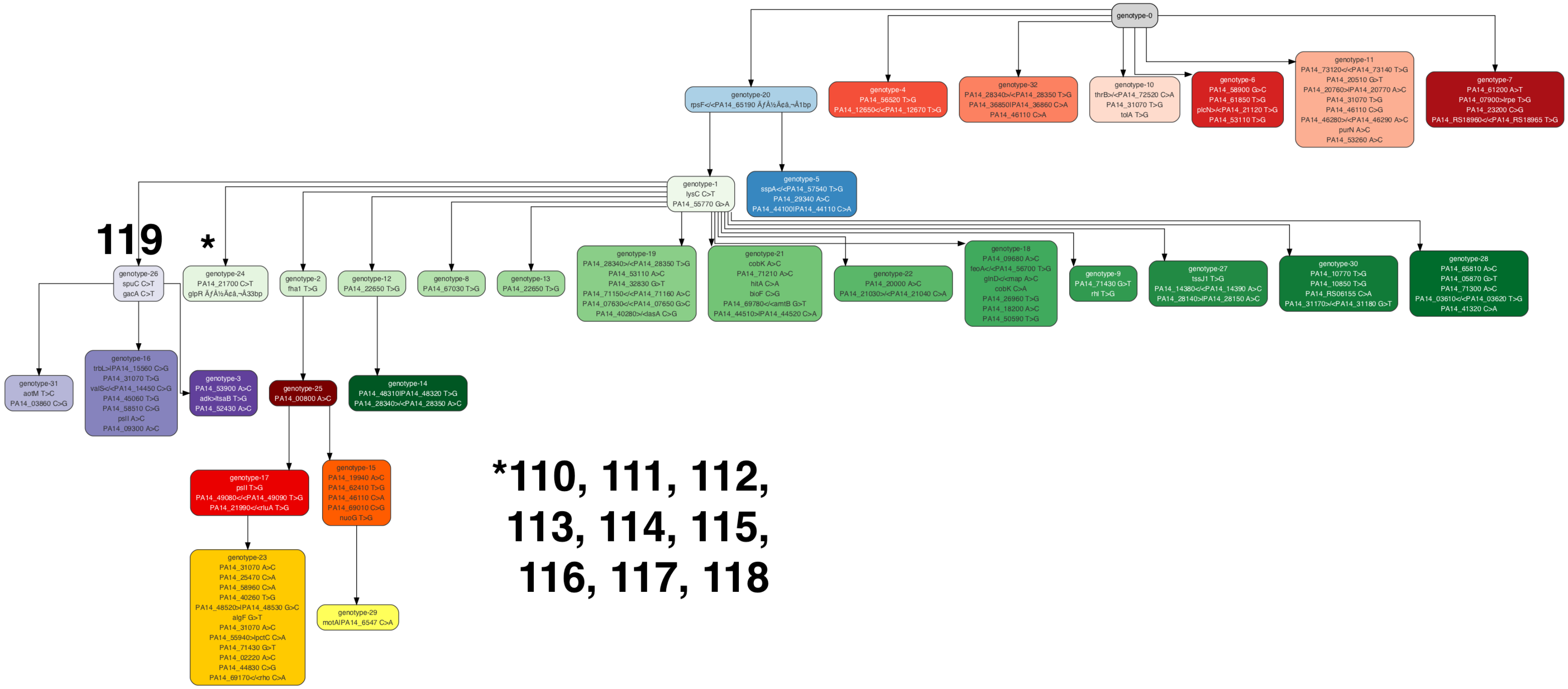

Figure S7 Ten clones isolated from the P1 population belong to two nodes on the P1 population ancestry. Colors denote ancestral lineages and do not correspond to any previous figures.

Table S4. All mutational frequencies that were determined to be significantly enriched in either biofilm or planktonic conditions during testing for ecological adaptations. Mutations correspond to red points in figure S8.
https://github.com/KatrinaHarris23/PALTEanalysis/blob/master/Table_S4.xlsx

	**PC1**	**PC2**	**PC3**
Vmax	-0.043	0.884	0.465
Motility	-0.695	-0.361	0.622
Bioiflm_production	0.717	-0.296	0.631

Table S5. Loadings are presented for the three variables that contributed to the PCA plot presented in Figure 1A.
